# Integrative analysis of the characteristic of lipid metabolism-related genes for the prognostic prediction of hepatocellular carcinoma: Retraction

**DOI:** 10.1097/MD.0000000000033332

**Published:** 2023-03-10

The article “Integrative analysis of the characteristic of lipid metabolism-related genes for the prognostic prediction of hepatocellular carcinoma,”^[1]^ which appeared in Volume 101, Issue 39 of *Medicine®* is being retracted due to duplicate submission and duplicate publication.^[2]^ The authors admitted the error and agreed to retract the article.
